# Oxygen‐Mediated Fe‐N_3_O Single‐Atom Sites Enabled by Lignin‐Derived Hierarchical Carbon for Efficient Oxygen Reduction

**DOI:** 10.1002/advs.78503

**Published:** 2026-11-09

**Authors:** Xuhong Zheng, Yukun Luo, Mengxuan Zhang, Xuebin Lv, Tianguang Wang, Jing Gu, Kang Xiao, Zhaoqing Liu, Haoran Yuan, Gaixiu Yang

**Affiliations:** ^1^ Key Laboratory of Biodiversity and Environment on the Qinghai‐Tibetan Plateau, Ministry of Education, School of Ecology and Environment Xizang University Lhasa P. R. China; ^2^ Guangzhou Institute of Energy Conversion Chinese Academy of Sciences (CAS) Guangzhou P. R. China; ^3^ Guangdong Provincial Key Laboratory of High‐Quality Recycling of End‐of‐Life New Energy Devices Guangzhou P. R. China; ^4^ School of Chemistry and Chemical Engineering Guangzhou University Guangzhou P. R. China

**Keywords:** asymmetric coordination, Fe single atom catalysts, lignin, oxygen reduction reaction, Zn‐air battery

## Abstract

Iron single‐atom catalysts (Fe SACs) offer excellent performance for the oxygen reduction reaction (ORR). However, their catalytic activity remains limited by the coordination structure and the accessibility of the active sites. Optimizing the local coordination and pore structure of the catalyst is imperative. Herein, we report a dual‐template strategy using oxygen‐rich lignin to synthesize Fe single‐atom catalysts with asymmetric Fe‐N_3_O coordination embedded in hierarchical porous carbon (HPC). Lignin serves both as the carbon precursor and a coordination modulator, while Zn/MgO dual templates facilitate the creation of a tri‐modal (micro/meso/macro) pore architecture. Operando spectroscopy and DFT calculations reveal that the asymmetric Fe‐N_3_O configuration downshifts the d‐band center of Fe, which weakens *OOH adsorption and accelerates charge transfer, leading to enhanced intrinsic activity. The optimized Fe‐N_3_O@HPC catalyst exhibits exceptional ORR activity in 0.1 M KOH, with a half‐wave potential of 0.87 V vs. RHE, surpassing Pt/C by 40 mV. Moreover, it exhibits significantly smaller decay over 30 h than commercial Pt/C. In zinc‐air batteries, the catalyst delivers a peak power density of 174.2 mW cm^−2^ and a specific capacity of 807 mAh g_Zn_^−1^. This research offers a cost‐effective methodology to design and fabricate high‐performance catalysts for ORR.

## Graphical abstract

A cooperative dual‐template strategy transforms oxygen‐rich lignin into hierarchical porous carbon hosting asymmetric Fe‐N_3_O single‐atom sites. The unique coordination downshifts the Fe d‐band center, weakening *OOH adsorption and accelerating charge transfer. The optimized catalyst delivers exceptional oxygen reduction activity (E_1/2_ = 0.87 V), outperforming commercial Pt/C.

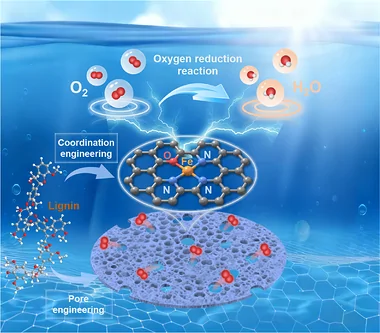

## 1 Introduction

The global transition toward renewable energy systems urgently demands cost‐effective electrocatalysts for the replacement of platinum‐group (Pt‐group) metals toward oxygen reduction reaction [1, 2]. Although Pt‐based catalysts (e.g., commercial Pt/C) exhibit superior catalytic activity, their practical application faces fundamental constraints including prohibitive costs and insufficient durability [3, 4, 5]. These limitations have accelerated the exploration of Pt group metal‐free alternatives [6, 7]. Fe‐N‐C single‐atom catalysts have emerged as leading candidates, offering near‐100% atomic utilization and tunable electronic structures [8]. Yet, their widespread adoption faces three fundamental challenges: (i) the difficulty in simultaneously achieving high metal loading and uniform coordination environments, (ii) limited understanding of complex coordination structure (e.g., Fe‐N_x_O_y_) in carbon supports, and (iii) poor mass transport in microporous‐dominated systems [9, 10, 11, 12].

To circumvent organic ligand limitations, recent efforts focus on utilizing natural biomass as sustainable carbon sources for catalyst preparation [4, 13, 14]. Among these, lignin, the important component of biomass, is a promising candidate with high carbon content (∼60 wt%) and unique amorphous polyphenolic network that provides abundant coordination sites and facilitates metal ion dispersion [15, 16]. Additionally, the conversion of lignin into SACs has obvious economic and environmental benefits because only about 5% of lignin is used in high‐value applications each year and most is discarded as a by‐product of the paper industry [16]. Currently, research on lignin‐derived SACs has already emerged in metal‐air batteries, pollutant degradation, and many other fields [15, 16, 17, 18]. However, the critical challenges remain for lignin‐derived SACs in pore structure engineering and coordination environment modulation due to the complex composition and inherent structure of lignin. Thus, the fabrication of hierarchical porous structures with micro‐, meso‐ and macropores remains to be studied. Furthermore, prevailing M‐N_4_ coordination configurations in lignin‐derived SACs reported in recent studies exhibit suboptimal electronic structures, especially in ORR process [19]. From the crystal‐field perspective, the Fe center in the perfect *D*_4h_ symmetric Fe‐N_4_ moiety features a characteristic 3*d* orbital splitting, resulting in a symmetric charge distribution around the metal center. The catalytic activity of Fe‐N_4_ toward ORR primarily originates from the hybridization between the Fe 3*d* orbitals (particularly *dz^2^* and *dyz/dxz*) and the π* orbitals of O_2_ [20, 21]. However, the planar symmetric coordination imposes a rigid d‐band center pinned close to the Fermi level, which leads to excessively strong adsorption of oxygenated intermediates [22, 23]. To circumvent these intrinsic limitations, a widely adopted strategy is to introduce heteroatoms into the first coordination sphere to construct an asymmetric coordination configuration [24, 25, 26]. This symmetry‐breaking approach effectively redistributes the local charge density and weakens the adsorption strength of intermediates, thus optimizing the overall reaction free‐energy landscape. Despite lignin's inherent oxygen‐rich phenolic structure, strategic incorporation of O atoms into Fe coordination environments remains synthetically challenging.

Herein, we present a cooperative dual‐template strategy that leverages lignin's molecular architecture to synthesize Fe SACs with asymmetric Fe‐N_3_O coordination and hierarchical porosity. Unlike conventional approaches relying on inert N‐rich precursors (e.g., ZIF‐8), our method exploits oxygen‐rich lignin as both a carbon matrix and an O‐donor to stabilize unusual Fe‐N_3_O sites, while Zn/MgO templates synergistically construct a tri‐modal pore system (micro/meso/macro). We reveal that volatile Zn generates micropores, whereas MgO templates create interconnected meso/macropores accelerating O_2_ diffusion. The optimized catalyst (Fe‐N_3_O@HPC) achieves a half‐wave potential of 0.87 V vs. RHE in alkaline media, outperforming Pt/C by 40 mV and maintaining 87% activity after 30 h. Operando electrochemical impedance spectroscopy uncovers a faster O_2_ adsorption kinetics. First‐principles calculations attribute this enhancement to the asymmetric Fe‐N_3_O configuration, which downshifts the d‐band center and optimizes *OOH adsorption energy. When integrated into zinc‐air batteries, Fe‐N_3_O@HPC delivers a peak power density of 174.2 mW cm^−2^ surpassing most reported non‐Pt group metal‐free catalysts, while enabling 807 mAh g_Zn_^−1^ specific capacity.

## 2 Results and Discussion

As illustrated in Figure 1a, the preparation of lignin‐derived hierarchical porous SAC (named as Fe‐N_3_O@HPC) was synthesized via a dual‐template strategy and the coordination between the metal precursor and phenolic hydroxyl groups of lignin with external N source. Lignin served as both the carbon and oxygen source. And melamine was used for the additional N‐doping agent. Lignin, Fe salt, Zn salt, nano MgO and melamine were thoroughly mixed in water, followed by solvent evaporation and carbonization at 910 °C. After etching, catalyst with Fe and N/O co‐coordinated structure was obtained. Lignin (as shown in Figure S1a,b), with its three‐dimensional amorphous structure contains abundant oxygen‐containing functional groups, such as hydroxyl and carboxyl groups, coordinates with transition metal ions (Fe^3+^ and Zn^2+^) to form supramolecular complexes. During this process Fe and Zn ions competitively interact with lignin and melamine through coordination bonding, where the predominant Zn^2+^ ions spatially separate Fe^3+^ ions, preventing their aggregation into metallic clusters during pyrolysis [27]. Concurrently, lignin undergoes continuous swelling in water during solvent evaporation, exposing additional oxygen‐containing functional groups. Meanwhile, the sheet‐like MgO (Figure S1c,d) template reacts with water to form Mg(OH)_2_ on the surface, which strongly hydrogen‐bonds with lignin's oxygen‐containing groups, enabling co‐assembly into the three‐dimensional network of lignin [28]. As lignin swelling progresses, the MgO templates diffuse into the mesoscale structure, ultimately inducing the generation of nanosheet architectures (Figure S1e,f). Subsequently, the obtained supramolecular complex precursor was subjected to carbonization. Under high temperature, Zn ions would be reduced by carbon substrate to form Zn atoms. And the Zn atoms could serve as soft templates and evaporate to form the pore structures. Meanwhile, MgO templates would be remained due to excellent thermal stability during carbonization and eventually removed by acid etching.

**FIGURE 1:**
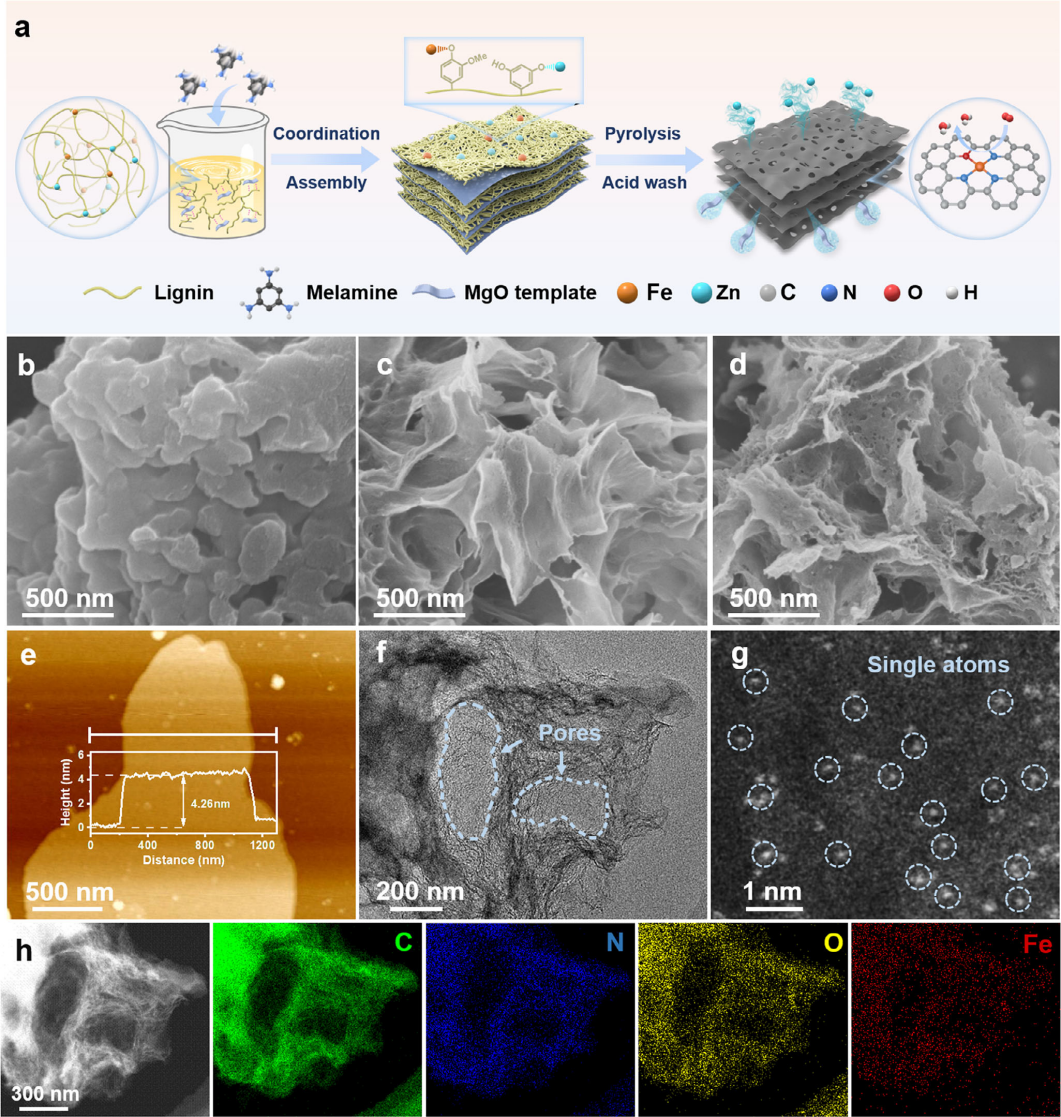
(a) Schematic illustration of the fabrication strategy for hierarchical porous Fe single atom catalyst (Fe‐N_3_O@HPC). SEM images of (b) Fe‐N/O@Micro‐PC, (c) Fe‐N/O@Meso/Macro‐PC and (d) Fe‐N_3_O@HPC. (e) AFM image, (f) TEM image, (g) AC‐HAADF‐STEM image and (h) EDS elemental mapping (C, N, O, Fe) of Fe‐N_3_O@HPC.

The distinct morphological differences of all the samples have been shown by the SEM images (Figure 1b–d and Figure S1g,h), which clearly indicated the critical roles of Zn ions/atoms and MgO templates in the generation of porous structure. As shown in Figure S1g,h, the control sample, nitrogen/oxygen‐doped carbon catalyst containing Fe element (Fe‐N/O@PC), prepared without Zinc acetate and MgO templates exhibited a low‐porous, surface‐flattened block‐like morphology. In contrast, the catalyst (Fe‐N/O@Micro‐PC) prepared with the addition of Zinc acetate, maintained its blocky architecture but developed surface roughness (Figure 1b), attributed to surface disruption caused by Zn volatilization during pyrolysis. Notably, as shown in Figure 1c, the catalyst (Fe‐N/O@Meso/Macro‐PC) obtained with MgO nanosheet as only templates, exhibited numerous 2D ultrathin nanosheet catalyst with hierarchical porous structure. Most significantly, the synergistic combination of Zinc acetate and MgO templates yielded Fe‐N_3_O@HPC with abundant porous nanosheets, as clearly observed in SEM images (Figure 1d). The thickness of nanosheets was also measured by atomic force microscopy (AFM). As depicted by Figure 1e, linear scan revealed that the nanosheets of Fe‐N_3_O@HPC had a thickness of approximately 4 nm, confirming its ultrathin graphene‐like structure.

To further investigate the effects of Zn atoms and MgO templates on pore structure evolution, N_2_ adsorption‐desorption experiments were conducted to analyze catalyst porosity, including pore size distribution and specific surface area. As shown in Figure S2a, both Fe‐N/O@Micro‐PC and Fe‐N/O@PC exhibited significant adsorption capacities at low relative pressures (P/P_0_< 0.02) with negligible hysteresis loops in the high‐pressure range (P/P_0_ = 0.4‐1.0), suggesting their predominantly microporous structures. Notably, Fe‐N/O@Micro‐PC demonstrated enhanced low‐pressure adsorption compared to Fe‐N/O@PC, illustrating the promoting effect of Zn atoms for the generation of micropores by acting as soft templates. This phenomenon can be attributed to the volatilization of Zn atoms at elevated temperatures (> 800°C). In comparison, MgO‐regulated catalysts (Fe‐N/O@Meso/Macro‐PC and Fe‐N_3_O@HPC) exhibited characteristic Type IV isotherms accompanied by H4‐type hysteresis loops at high relative pressures. The observed hysteresis loops arise from capillary condensation within mesopores, confirming the critical role of MgO templates in promoting mesopore formation and hierarchical porous architectures. Furthermore, the significantly enhanced low‐pressure adsorption capacity of Fe‐N_3_O@HPC relative to Fe‐N/O@Meso/Macro‐PC concurrently highlights the synergistic microporosity‐enhancing effect of Zn atoms. The pore size distribution profiles analyzed by DFT method (Figure S2b) revealed distinct structural characteristics among the catalysts. Both Fe‐N/O@Micro‐PC and Fe‐N/O@PC exhibited micropore‐dominated architectures with pore diameters ranging 0.6 to 1.6 nm, while Fe‐N/O@Micro‐PC demonstrated significantly enhanced microporosity compared to Fe‐N/O@PC. In contrast, the MgO‐modified Fe‐N/O@Meso/Macro‐PC was characterized by the presence of mesopores (2‐50 nm) and macropores (centered at ∼100 nm). Under the combined interaction of Zn atoms and MgO templates, Fe‐N_3_O@HPC catalyst exhibited hierarchical porous structure with a Brunauer‐Emmett‐Teller (BET) specific surface area of 1078 m^2^ g^−1^ and a total pore volume of 0.86 cm^3^ g^−1^ (Figure S2c,d). These values significantly surpass those of Fe‐N/O@Meso/Macro‐PC (567 m^2^ g^−1^, 0.63 cm^3^ g^−1^), Fe‐N/O@Micro‐PC (325 m^2^ g^−1^, 0.2 cm^3^ g^−1^) and Fe‐N/O@PC (158 m^2^ g^−1^, 0.097 cm^3^ g^−1^). The unique nanosheet structure featured with tri‐modal (micro/meso/macro) pore architecture are favorable to guarantee the exposure of internal active sites and enable fast mass transfer [29].

XRD patterns and Raman spectra were analyzed to systematically investigate structural and compositional variations among the samples. The XRD profiles (Figure S3) exhibited two characteristic broad diffraction peaks at 25° and 45° across all samples, corresponding to the C (002) and (101) crystallographic planes, respectively [30, 31]. Notably, Fe‐N/O@PC displayed additional low‐intensity peaks consistent with Fe_3_O_4_ (PDF#86‐1350), suggesting metallic phase formation through iron aggregation during carbonization in the absence of Zn atoms and MgO templates. This observation confirms the critical role of Zn atoms and MgO in suppressing metal nanoparticle formation through atomic dispersion stabilization. Moreover, Raman spectra (Figure S4) revealed two characteristic vibration modes: the defect‐associated D‐band (1335 cm^−1^) and the graphitic‐associated G‐band (1580 cm^−1^). The graphitization degree of the obtained catalysts was estimated through the peak intensity ratio of the D‐band and G‐band (I_D_/I_G_) [32]. Quantitative evaluation through the intensity ratio (I_D_/I_G_) demonstrated enhanced structural ordering in Zn‐regulated samples: Fe‐N_3_O@HPC (1.04) and Fe‐N/O@Micro‐PC (1.01) exhibited significantly lower ratios compared to Fe‐N/O@Meso/Macro‐PC (1.17) and Fe‐N/O@PC (1.22). This reduction in I_D_/I_G_ ratios indicates that Zn‐mediated thermal processing effectively mitigated structural defects while promoting graphitic domain development, which could be attributed to the catalytic role of Zn ions in promoting dehydration and aromatization reactions at low temperatures and the stress/catalytic effects induced by Zn^2+^ reduction and Zn evaporation at high temperatures [33, 34].

Transmission electron microscopy (TEM) was carried out to further determine the detailed morphology characteristics. TEM images (Figure 1f) showed that the as‐prepared Fe‐N_3_O@HPC exhibited a two‐dimensional sheet‐like morphology with abundant pore structures. And high‐resolution transmission electron microscopy (HR‐TEM) image of Fe‐N_3_O@HPC (Figure S5) clearly showed the lattice fringe of the graphene sheets. Fe‐N/O@Meso/Macro‐PC (Figure S6) also presented a two‐dimensional nanosheet morphology while Fe‐N/O@Micro‐PC displayed a block‐like morphology (Figure S7). No Fe particles were appeared in the randomly selected HR‐TEM images of Fe‐N_3_O@HPC, Fe‐N/O@Meso/Macro‐PC or Fe‐N/O@Micro‐PC, indicating that the Fe atoms are uniformly dispersed. In contrast, Fe‐N/O@PC exhibited metallic nanoparticles in TEM images with obvious 0.254 nm lattice fringe on HR‐TEM (Figure S8).

The aberration‐corrected high‐angle annular dark‐field scanning transmission electron microscope (AC‐HAADF‐STEM) was employed to further investigate the atomic dispersion of monatomic Fe within the Fe‐N_3_O@HPC. As shown in Figure 1g, Fe single atoms were evenly distributed in the carbon matrix with no observable aggregation phenomenon detected, confirming their atomically isolated distribution. The distribution of other atoms was also analyzed by energy‐dispersive X‐ray (EDS) mapping. As illustrated in Figure 1h, the uniformly distributed of Fe, N, O, and C were evidenced in the EDS mapping of Fe‐N_3_O@HPC.

Likewise, above elements were also uniformly dispersed within the Fe‐N/O@Meso/Macro‐PC and Fe‐N/O@Micro‐PC from the EDS mapping images (Figures S6 and S7). However, the locally high signal density can be observed in EDS Fe mapping image of Fe‐N/O@PC (Figure S8), verifying significant aggregation of Fe atoms. This further confirmed that Fe atoms aggregate during pyrolysis in the absence of Zn ions and MgO templates.

X‐ray photoelectron spectroscopy (XPS) survey spectrum (Figure S9) revealed the coexistence of C, N, and O elements in the near‐surface region. Notably, no distinct Fe signals were observed in either the survey spectrum or the high‐resolution Fe 2*p* spectra (Figure S10). However, the existence of Fe can be confirmed by the Inductively coupled plasma‐Mass Spectrometry (ICP‐MS), and their content are 0.5841%, 0.5989%, 0.7768% and 1.4818% (Table S1), respectively. This could be ascribed to the ultralow Fe content and the limited probing depth of XPS (∼5‐10 nm) [35].

The high‐resolution N 1*s* XPS spectra (Figure 2a and Figure S11) for the samples were deconvoluted into five distinct components: pyridinic N (398.23 eV), Fe‐N_x_ (398.99 eV), pyrrolic N (399.72 eV), graphitic N (401.19 eV), and oxidized N (404.19 eV) species [36]. Among these N species, the pyrrolic N have been proven to possess a high 2e^−^ ORR selectivity due to its optimal adsorption strength for *OOH species [37, 38]. On the other hand, pyridinic N and Fe‐N_x_ are widely regarded as active sites for the 4e^−^ ORR pathway [3, 39]. Intriguingly, Fe‐N/O@PC exhibited a significantly higher pyrrolic N content (37.6%) compared to its Fe‐N_x_ content (7.3%), likely due to Fe aggregation during synthesis. As specific surface area and pore volume increase, the relative abundance of Fe‐N_x_ and pyridinic N gradually increased, accompanied by a corresponding decrease in pyrrolic N. This correlation suggests that pore structure modulation directly influences the coordination environment of nitrogen species, thereby governing the ORR pathway selectivity. Additionally, the high‐resolution O 1*s* spectra of all samples (Figure S12) exhibited prominent Fe‐O bonding signals, indicating the coexistence of oxygen‐coordinated Fe species in the final catalysts [25, 35]. The functional groups on the surfaces were also tested by Fourier transform infrared (FT‐IR) spectroscopy and the results were shown in Figure S13. All samples showed similar functional groups, indicating that the differences on the surfaces originate from morphology and pore structure.

**FIGURE 2:**
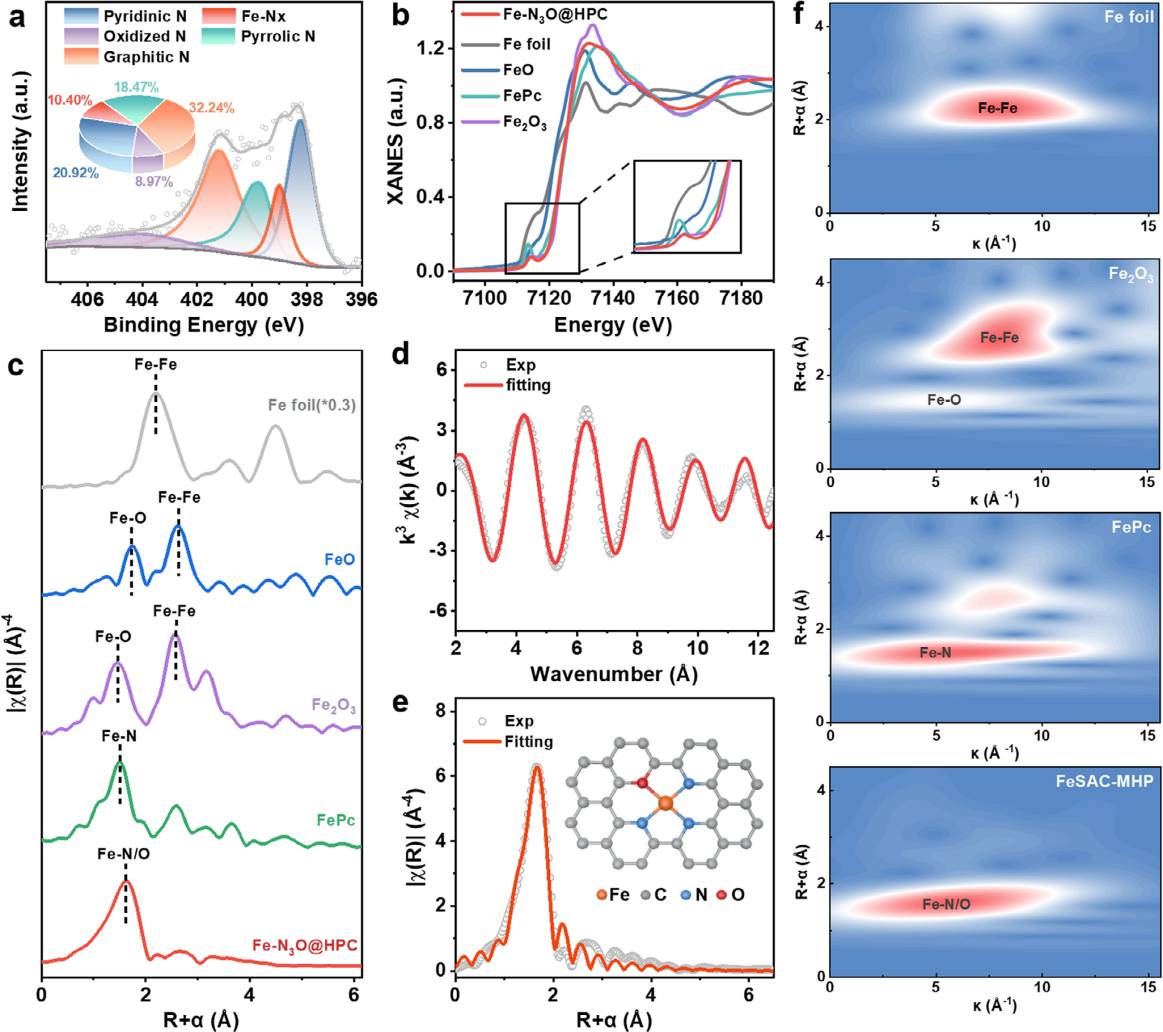
(a) XPS high‐resolution spectra of the N 1*s* of Fe‐N_3_O@HPC. (b) Fe K‐edge X‐ray absorption near‐edge structure spectra. (c) FT k^3^‐weighted EXAFS spectra. FT‐EXAFS fitting in (d) R space and (e) k space of Fe‐N_3_O@HPC. (f) Wavelet‐transforms for the k^3^‐weighted Fe K‐edge EXAFS signals for Fe‐N_3_O@HPC, and the reference samples of bulk Fe foil, Fe_2_O_3_ and FePc.

X‐ray absorption spectroscopies (XAS) were used to elucidate the coordination environment of monatomic Fe catalysts in Fe‐N_3_O@HPC. As depicted in the X‐ray absorption near‐edge structure spectra (XANES) of Fe K‐edge (Figure 2b and Figure S14), the near‐edge energy adsorption threshold of Fe‐N_3_O@HPC was located between the Fe_2_O_3_ and FeO, implying an intermediate oxidation state of Fe between +2 and +3 [40]. Meanwhile, the XANES profile of Fe‐N_3_O@HPC closely resembles that of iron phthalocyanine (FePc), a model compound with a square‐planar Fe‐N_4_ configuration. Both spectra exhibit a characteristic pre‐edge peak at 7110–7120 eV, associated with the 1*s* → 4*p_z_* electronic transition typically observed in planar Fe‐N_4_ coordination systems [41, 42]. The Fourier‐transformed (FT) k^3^‐weighted χ(k)‐function Fe K‐edge extended X‐ray absorption fine structure spectra (EXAFS) provided particular information about the local coordination of Fe sites (Figure 2c). The absence of a Fe‐Fe scattering path at ∼2.2 Å (characteristic of metallic Fe or Fe nanoparticles) confirms the atomic dispersion of Fe species in Fe‐N_3_O@HPC [42]. A dominant peak at ∼1.6 Å corresponds to the first coordination shell of Fe‐N bonds, closely matching the Fe‐N distance in FePc (1.5 Å) [43]. However, a subtle positive shift (∼0.1 Å) in the Fe‐N_3_O@HPC spectrum suggests additional scattering contributions, likely from oxygen ligands [25]. Combined with XPS evidence and known abundance of oxygen‐containing functional groups in the lignin‐derived support, this shift strongly implies the formation of Fe‐O bonds. Quantitative EXAFS fitting (Figure 2d,e and Table S2) further verifies a coordination structure consisting of three nitrogen atoms and one oxygen atom (Fe‐N_3_O). In addition, Wavelet transform EXAFS analysis (Figure 2f) provides complementary spatial resolution in both k‐ and R‐space. The maximum intensity for Fe‐N_3_O@HPC occurs at ∼5.7 Å^−1^, distinct from those of Fe foil (8.1 Å^−1^) and Fe_2_O_3_ (8.0 Å^−1^), ruling out Fe‐Fe metallic contributions. The Wavelet transform contour profile closely aligns with FePc (5.8 Å^−1^), consistent with predominant Fe‐N bonding. The slight energy shift further supports the coexistence of Fe‐N and Fe‐O coordination paths. Collectively, XAS analyses unambiguously identify the atomic dispersion of Fe in HPC with a Fe‐N_3_O coordination structure. Different from traditional symmetric electron distribution Fe‐N_4_ structure, which tends to cause strong adsorption of intermediates, the formation of Fe‐N_3_O asymmetric sites could prevent excessive adsorption of intermediates during catalysis, thereby ensuring high catalyst activity.

To evaluate the ORR electrocatalytic performance, cyclic voltammetry (CV) and linear scanning voltammetry (LSV) analyses were systematically performed on Fe‐N_3_O@HPC, Fe‐N/O@Meso/Macro‐PC, Fe‐N/O@Micro‐PC, Fe‐N/O@PC and commercial Pt/C catalysts using a standard three‐electrode system in 0.1 M KOH electrolyte. The CV profiles acquired under inert N_2_ atmosphere (Figure S15) showed negligible oxygen reduction signals for all samples, whereas distinct cathodic peaks emerged in O_2_‐saturated conditions. Notably, Fe‐N_3_O@HPC demonstrated the most positive oxygen reduction peak potential (0.81 V vs. RHE), surpassing Pt/C (0.80 V), Fe‐N/O@Meso/Macro‐PC (0.79 V), Fe‐N/O@Micro‐PC (0.77 V), and Fe‐N/O@PC (0.74 V). LSV measurements at 1600 rpm provided further insights into ORR activities. As shown in Figure 3a, Fe‐N/O@PC exhibited inferior performance without a significant diffusion‐limited current plateau, attributable to its low surface area and non‐porous architecture. In contrast, the hierarchically porous Fe SACs derived from lignin precursors exhibited progressively enhanced ORR performance with increasing surface area and pore structure complexity. Among these, Fe‐N_3_O@HPC achieved exceptional metrics with an onset potential (E_onset_) of 0.98 V and a half‐wave potential (E_1/2_) of 0.87 V, exceeding those of Pt/C (0.95 V/0.83 V), Fe‐N/O@Meso/Macro‐PC (0.94 V/0.82 V), and Fe‐N/O@Micro‐PC (0.89 V/0.72 V). The enhanced reaction kinetics of Fe‐N_3_O@HPC were corroborated by its Tafel slope of 80 mV dec^−1^ (Figure 3b), lower than those of Pt/C (87 mV dec^−1^), Fe‐N/O@Meso/Macro‐PC (89 mV dec^−1^), Fe‐N/O@Micro‐PC (110 mV dec^−1^), and Fe‐N/O@PC (178 mV dec^−1^). This kinetic advantage was further quantified through analysis of the mass‐transport‐corrected current densities. Remarkably, as shown in Figure 3c, Fe‐N_3_O@HPC delivered a remarkable kinetic current density of 26.3 mA cm^−2^ at 0.8 V, representing 1.8‐fold, 3.5‐fold, and 23.9‐fold enhancements over Pt/C (14.6 mA cm^−2^), Fe‐N/O@Meso/Macro‐PC (7.6 mA cm^−2^) and Fe‐N/O@Micro‐PC (1.1 mA cm^−2^), respectively. Meanwhile, the Fe mass‐normalized activity of Fe‐N_3_O@HPC reaches 17.65 A g_Fe_^−1^, representing 3.6‐fold, and 31.5‐fold enhancements over Fe‐N/O@Meso/Macro‐PC (4.97 A g_Fe_^−1^) and Fe‐N/O@Micro‐PC (0.56 A g_Fe_^−1^), respectively (Figure S16). The remarkable mass activity of Fe–N_3_O@HPC further demonstrates that the formation of atomically dispersed Fe single‐atom sites and the hierarchical porous structure effectively promote the intrinsic activity of the catalyst.

**FIGURE 3:**
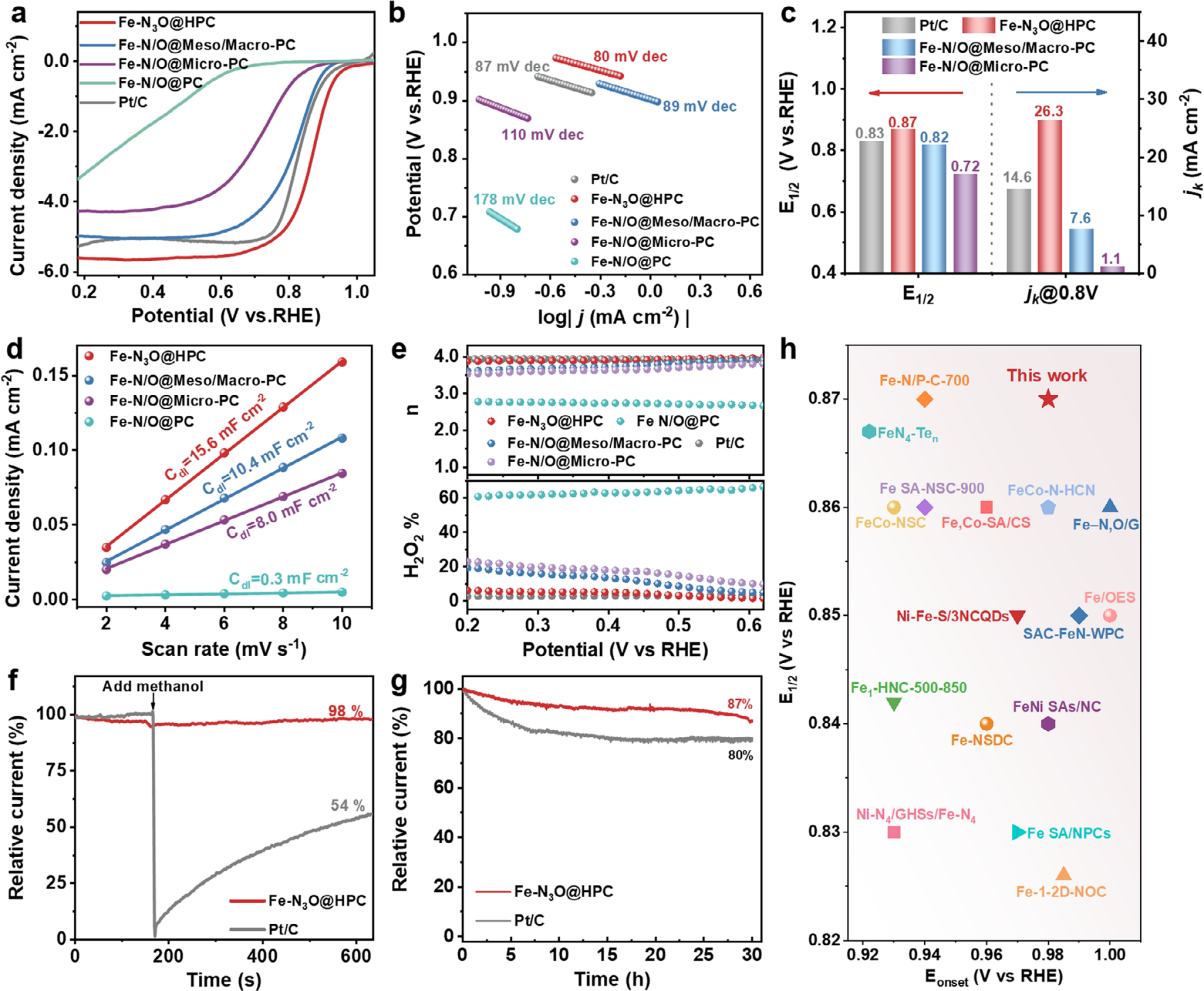
(a) LSV curves collected at 10 mV s^−1^ with 1600 rpm. (b) corresponding Tafel slopes. (c) comparison of E_1/2_ and *j_k_@0.8 V*. (d) C_dl_ curves. (e) Electron transfer number (n) and peroxide (H_2_O_2_) yield calculated by rotating ring‐disk electrode (RRDE) measurements. (f) Current density retention curves after the addition of 1 mL methanol in O_2_‐saturated 0.1 M KOH. (g) Normalized i‐t curves from long‐term chronoamperometry tests at 0.4 V vs. RHE under a rotating rate of 1600 rpm. (h) Comparison of E_onset_ and E_1/2_ for Fe‐N_3_O@HPC and reported Fe‐based catalysts in other literatures.

Furthermore, the electrochemical active specific surface areas (ECSA)‐a critical parameter reflecting the electrode‐electrolyte interface through double‐layer capacitance (C_dl_) were measured at varying scan rates to characterize the surface performance of the obtained catalyst (Figure S17). As shown in Figure 3d, the Fe‐N/O@PC sample exhibited a significantly lower C_dl_ value compared to other catalysts, which can be attributed to its insufficient pore architecture and reduced specific surface area. With the introduction of templating agents, we observed a progressive enhancement in C_dl_ values from Fe‐N/O@Micro‐PC to Fe‐N_3_O@HPC, directly correlated with the developed specific surface area and optimized hierarchical pore structures. Eventually, Fe‐N_3_O@HPC exhibited the highest C_dl_ value of 15.6 mF cm^−2^ and the largest ECSA of 76.5 m^2^ g^−1^, indicating superior mass transport capabilities and enhanced utilization of active sites.

RRDE and RDE tests at various rotation speeds from 625 to 2500 rpm were also performed to monitor the H_2_O_2_ yield and electron transfer number. Due to its higher pyrrolic N content and relatively low specific surface area, the Fe‐N/O@PC exhibited partial two‐electron pathway selectivity. With the progressive increase in specific surface area and pore volume, the four‐electron pathway became progressively dominant. Thus, the Fe‐N_3_O@HPC demonstrated remarkably low H_2_O_2_ yields (< 5%) throughout the potential range of 0.2‐0.6 V vs. RHE (Figure 3e). After the correction of collection efficiency (Figure S18), the calculated electron transfer number of 3.9 for Fe‐N_3_O@HPC approaches the theoretical value for an ideal four‐electron pathway (n = 4.0), demonstrating its exceptional catalytic selectivity. This conclusion was further corroborated by Koutecký‐Levich (K‐L) analysis (Figure S19), which yielded an average electron transfer number approaching 4.0.

The exceptional catalytic stability of Fe‐N_3_O@HPC was further validated through comprehensive testing. The methanol tolerance was tested by injecting methanol (1 mL) into the electrolyte during chronoamperometric measurements at 0.4 V (Figure 3f). Remarkably, Fe‐N_3_O@HPC exhibited negligible current decay (< 5%) upon methanol introduction, whereas the Pt/C catalyst suffered a rapid performance decline, retaining only 54% of its initial current after 600 s. This superior methanol resistance highlighted Fe‐N_3_O@HPC's practical advantages for fuel cell applications where fuel crossover may occur. To confirm the dominant role of Fe single‐atom sites in ORR catalysis, KSCN poisoning experiment was conducted. Introduction of 10 mM KSCN into the electrolyte caused significant degradation in both half‐wave potential (ΔE_1/2_ = 30 mV) and limiting current density (8% reduction), as evidenced by LSV measurements (Figure S20). This strong poisoning effect explicitly identifies atomic Fe centers as the primary active sites, consistent with previous mechanistic studies [44]. Long‐term stability assessments further demonstrated Fe‐N_3_O@HPC's robustness. Chronoamperometric testing at 0.4 V over 30 h revealed 87% current retention, outperforming Pt/C's 80% retention under identical conditions (Figure 3g). XPS analysis was performed on the catalyst before and after the stability test. As shown in Figure S21, there is no significant difference in XPS spectra of Fe‐N_3_O@HPC before and after chronoamperometric testing. To circumvent the limitation the weak signal of Fe element, ICP‑MS analysis on the electrolyte after the long‑term test was also conducted, the results show that the amount of dissolved Fe is 1.097 µg L^−^^1^, corresponding to a Fe loss of 2.5% (Table S3). This confirms that no significant demetallation occurs. In order to further evaluate the stability in complex working environments, ADT measurements through CV cycles between 0.6 and 1.0 V vs. RHE were also conducted, which involve harsher testing conditions than i‐t test and simultaneously triggering multiple degradation mechanisms such as carbon corrosion, denitrogenation and pore structure collapse. The results demonstrate that Fe–N_3_O@HPC exhibits smaller degradation in both half‑wave potential and limiting current density, indicating its structural stability compared to Pt/C. (Figure S22). Notably, Fe‐N_3_O@HPC's ORR performance surpasses most reported Fe‐based catalysts and non‐precious metal catalysts in alkaline media (0.1 M KOH), as evidenced by comparative analysis of half‐wave potentials and onset potential (Figure 3h and Table S4). This combination of high activity, methanol tolerance, and operational stability positions Fe‐N_3_O@HPC as a highly promising contender for next‐generation energy conversion devices.

The operando electrochemical impedance spectroscopy (EIS) was conducted to elucidate the correlation between mass transfer dynamics and pore structural characteristics of the obtained catalysts during ORR process. As depicted in Figure 4a–d, phase angle plots of Fe‐N_3_O@HPC, Fe‐N/O@Meso/Macro‐PC, and Fe‐N/O@Micro‐PC catalysts exhibited two distinct peaks, corresponding to two characteristic time constants in Bode plots. The high‐frequency time constant was attributed to charge transfer processes at the electrode‐electrolyte interface, while the low‐frequency constant originated from mass transport limitations involving oxygen reactants and reaction intermediates/products [45, 46]. Notably, Fe‐N/O@PC displayed an additional phase angle peak in the intermediate frequency range (10^0^ ‐10^2^ Hz), absent in other catalysts. This feature likely reflects competing reaction pathways, as supported by the significantly higher two‐electron ORR selectivity of Fe‐N/O@PC compared to other samples [47]. The emergence of this intermediate‐frequency peak suggests dynamic competition between two‐electron and four‐electron ORR pathways under operational conditions. In the high‐frequency domain, all catalysts exhibited similar potential‐dependent EIS behavior. Increasing applied potentials caused a systematic decrease in phase angle magnitude accompanied by peak frequency upshifts. These effects showed pronounced enhancement with increasing specific surface area and pore volume, directly demonstrating the critical influence of catalyst porosity on mass transport efficiency. The observed correlation between structural parameters and impedance characteristics provides quantitative evidence that optimized pore architectures can effectively mitigate mass transfer limitations during ORR processes.

**FIGURE 4:**
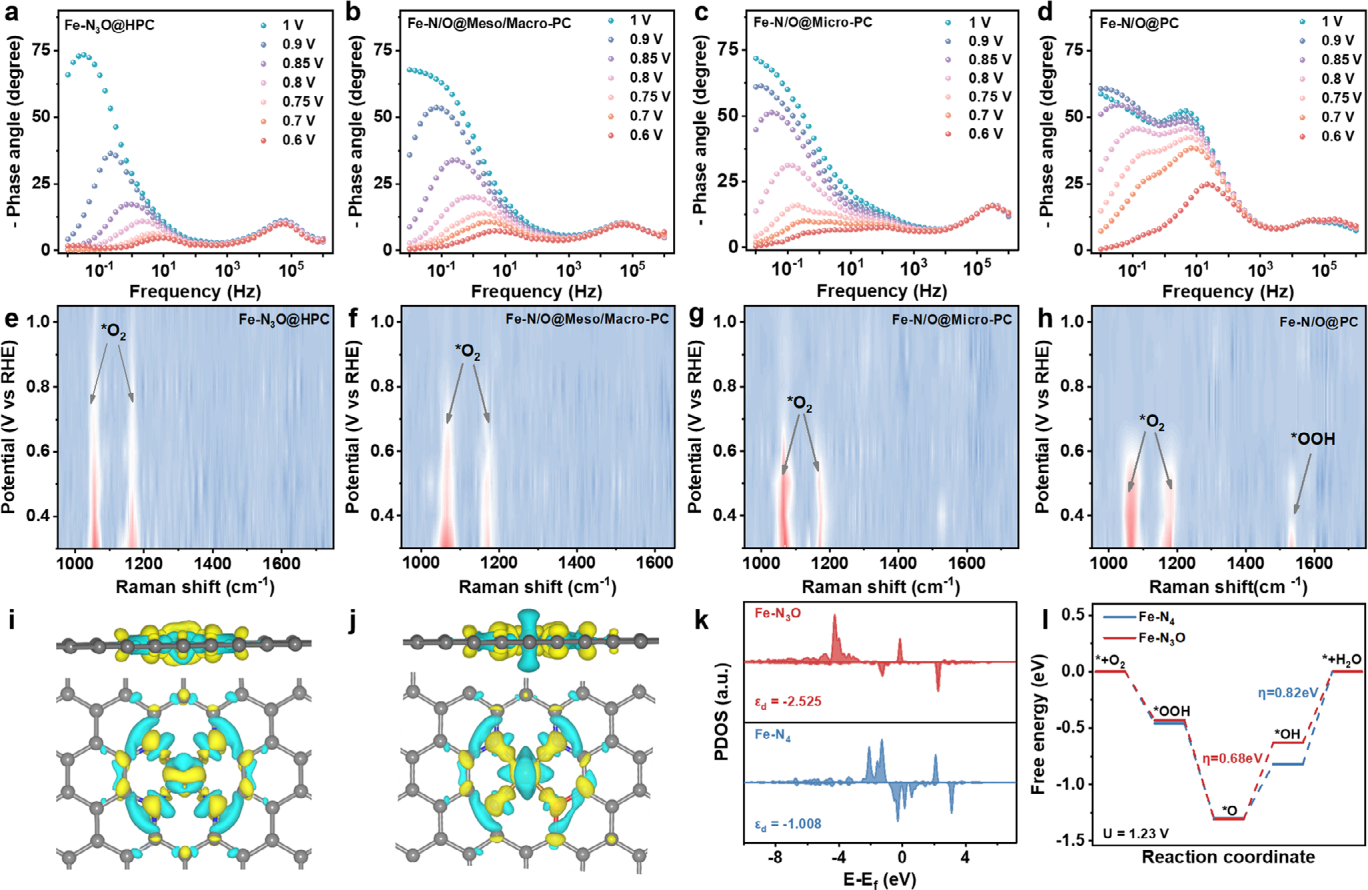
(a‐d) Bode plots from operando electrochemical impedance spectra and (e‐h) in‐situ Raman spectra for Fe‐N_3_O@HPC, Fe‐N/O@Meso/Macro‐PC, Fe‐N/O@Micro‐PC and Fe‐N/O@PC (i‐j) Charge density difference diagrams of Fe‐N_4_ site and Fe‐N_3_O site (k) PDOS (l) ORR free energy diagrams under U = 1.23 V of Fe‐N_4_ site and Fe‐N_3_O site.

Moreover, the Nyquist plots were fitted using equivalent circuit models to elucidate the mass transport properties of the catalysts, as shown in Figure S23. For Fe‐N_3_O@HPC, Fe‐N/O@Meso/Macro‐PC, and Fe‐N/O@Micro‐PC, a two‐time‐constant circuit model was employed containing solution resistance (R_s_), charge transfer resistance (R_ct_), mass transfer resistance (R_mt_), and constant phase angle element (CPE). In contrast, a three‐time‐constant model was required to fit Fe‐N/O@PC due to the emergence of an additional phase angle characteristic of the two‐electron pathway [48, 49]. Key observations revealed that R_mt_ decreased significantly with increasing applied bias potential, indicating enhanced oxygen and product diffusion. Notably, Fe‐N_3_O@HPC demonstrated both smaller R_mt_ values and a steeper potential‐dependent R_mt_ values reduction compared to other samples. This behavior highlights the advantageous role of its hierarchical pore structure in facilitating O_2_ transportation to the active site. Furthermore, Fe‐N_3_O@HPC exhibited consistently lower R_ct_ values across various potentials relative to Fe‐N/O@Meso/Macro‐PC and Fe‐N/O@Micro‐PC (Figure S24). This enhanced charge transfer performance can be attributed to the interconnected micro‐mesoporous architecture, which promotes both accessibility of active sites and efficient interfacial charge transport while maximizing utilization of Fe single atoms. To further resolve the composite impedance of electrochemical reaction and mass transfer, the DRT analysis was employed to evaluate electrochemical processes. The characteristic peaks in the high‐, mid‐, and low‐frequency regions could be corresponding to ion diffusion, ORR kinetics, and gas diffusion, respectively [50]. Notably, ion diffusion impedance is much lower than that of ORR and gas diffusion. As shown in Figure S25, The DRT spectra of all samples exhibit two distinct characteristic peaks in the low‐frequency and medium‐to‐high‐frequency regions, which can be assigned to the mass‐transport process and the charge‐transfer process, respectively. The peak of Fe‑N/O@PC between medium‐to‐high‐frequency region undergoes a clear splitting into two sub‐peaks. This splitting arises from the competitive coexistence of the 2e^−^ and 4e^−^ ORR pathways on Fe‑N/O@PC. Moreover, Fe‐N_3_O@HPC exhibits significantly accelerated charge transfer and mass transfer kinetics compared to Fe‐N/O@Meso/Macro‐PC, Fe‐N/O@ Micro‐PC, and Fe‑N/O@PC, as evidenced by the more pronounced shifted frequency and the sharper decreased amplitude of the DRT peak. This identifies the enhanced intrinsic charge‐transfer and mass‐transfer kinetics resulting from the optimization of pore structure and the formation of single‐atom active sites.

In order to elucidate the differences in reaction intermediates, in situ Raman spectroscopy was employed to monitor the ORR process (Figure 4e–h). Two broad Raman bands at 1000–1200 cm^−1^ were observed in Fe‐N_3_O@HPC, Fe‐N/O@Meso/Macro‐PC and Fe‐N/O@Micro‐PC, corresponding to the O‐O stretching vibration of *O_2_ species [51, 52]. Specifically, the band centered at ∼ 1064 cm^−1^ was attributed to molecular O_2_ adsorbed on the Fe sites, whereas the peak at ∼ 1160 cm^−1^ arose from O_2_ adsorption on the C‐N sites [53]. Notably, Fe‐N/O@PC exhibited an additional peak at about 1520 cm^−1^, which was assigned to the *OOH intermediate adsorbed on C‐N sites [54, 55] The distinct presence of the *OOH intermediate in Fe‐N/O@PC may stem from competitive two‐electron and four‐electron ORR pathways, likely influenced by its high pyrrolic N content and limited specific surface area. Comparative analysis of Raman band intensities within the 1000–1200 cm^−1^ region revealed enhanced oxygen adsorption signals at higher applied potentials for catalysts with larger specific surface areas and pore volumes. This correlation underscores the critical role of microporous hierarchical structures in preserving active sites and facilitating oxygen adsorption. In summary, the incorporation of hierarchical porosity, particularly micropore‐rich architectures, significantly enhances oxygen adsorption capacity and mass transfer kinetics in Fe‐N_3_O@HPC. These structural advantages synergistically contribute to its superior ORR catalytic activity.

Considering the contribution of Fe SAC coordination structure to ORR process, density functional theory (DFT) calculations were carried out to investigate the differences between Fe‐N_3_O and conventional Fe‐N_4_ structure in terms of microenvironment and catalytic mechanism. Optimized models were constructed for the Fe SACs based on information acquired in experimental assessments (Figure S26). Charge density difference analysis was performed to reveal the regulatory effect of oxygen atom on the electronic structure of Fe SAC. As exhibited in Figure 4i,j, the yellow color area can be assigned to the accumulation of electrons, while the blue color area represents their dissipation region. Compared to Fe‐N_4_ model, more pronounced electron depletion on the central Fe sites of Fe‐N_3_O structure, suggesting that the displacement of the coordinated N atom by an O atom in the Fe‐N_4_ site can facilitate charge redistribution at the central Fe atom. This result can be attributed to the higher electronegativity of the O atoms. Subsequently, the projected density of states (PDOS) and the d‐band centers (ɛ_d_) was further analyzed (Figure 4k). The ɛ_d_ values of Fe‐N_4_ and Fe‐N_3_O sites were ‐1.008 and ‐2.525, respectively. Compared to Fe‐N_4_ site, the downward shift of the d‐band centers of Fe atoms in Fe‐N_3_O implies that more antibonding orbitals are below the Fermi energy level, which facilitates the mitigation of the excessive adsorption of the oxygen‐containing intermediates on typical Fe‐N_4_ configuration during ORR [56]. The Gibbs free energy (ΔG) diagrams for Fe‐N_4_ and Fe‐N_3_O sites were investigated to further confirm difference in adsorption strength of the oxygen‐containing intermediates. The adsorption configurations of the oxygen‐containing intermediates (*O_2_, *OOH, *O, *OH) on Fe sites during ORR process are depicted in Figure S27. The Gibbs free energy diagram shows that all the elementary steps on the Fe‐N_3_O and Fe‐N_4_ sites are thermodynamically favorable at U = 0 V (Figure S28). Significantly, adsorption strengths of intermediates (*OOH and *OH) on the Fe‐N_3_O site are weaker than those of Fe‐N_4_ sites, indicating that the formation of asymmetric coordination of Fe‐N_3_O can effectively adjust the adsorption energy and improve the excessive adsorption of the intermediate at the Fe‐N_4_ site. As demonstrated in Figure 4l, the Gibbs free energy diagram at U = 1.23 V provides further insights into the impact of asymmetric coordination environment on the rate‐determining step (RDS). The RDS for Fe‐N_4_ is identified as the formation of H_2_O from *OH, with an energy barrier of 0.82 eV. In contrast, on the Fe‐N_3_O surface, the RDS shifts to the transformation of *O to *OH with a lower overpotential of 0.68 eV. Therefore, compared to Fe‐N_4_, the introduction of an oxygen atom in coordination structure not only aids in weakening the adsorption energy of ORR intermediates, but also results in a shift of RDS, effectively enhancing the intrinsic ORR activity. Overall, the substitution of an oxygen atom for a nitrogen atom in the Fe‐N_4_ site profoundly influences the electronic structure and orbital interactions, thereby fine‐tuning the adsorption free energy of ORR intermediates on the Fe site and consequently enhancing its intrinsic activity.

Motivated by its superior ORR activity, Fe‐N_3_O@HPC was employed as the cathode catalyst in a rechargeable Zn–air battery (ZAB), as schematically illustrated in Figure 5a. As shown in Figure S29, the Fe‐N_3_O@HPC‐based ZAB exhibited an open‐circuit voltage of 1.55 V, surpassing that of the Pt/C‐catalyzed counterpart (1.52 V). Notably, the voltage profiles under varying current densities (Figure 5b) revealed that Fe‐N_3_O@HPC consistently delivered higher operational voltages than Pt/C, with the performance disparity amplifying at elevated current densities. The sharp voltage drop of Pt/C at high current density can be attributed to water flooding arising from the local electrolyte infiltration induced by the substantial generation of OH^−^, which blocks oxygen transport. In contrast, the hierarchical porous structure of Fe–N_3_O@HPC ensures mass transfer and gas diffusion, sustaining its performance under high‑load conditions. This trend underscores the superior kinetics and mass transport properties of Fe‐N_3_O@HPC. Further evaluation of power output demonstrated that the Fe‐N_3_O@HPC‐based ZAB achieved a remarkable peak power density of 174.2 mW cm^−2^ at 250 mA cm^−2^ (Figure 5c), significantly exceeding the Pt/C‐based system (146.9 mW cm^−2^ at 181.5 mA cm^−2^) and outperforming state‐of‐the‐art catalysts documented in recent literature (Table S5). Practical applicability was confirmed through successful operation of a digital timekeeping device powered by the Fe‐N_3_O@HPC‐based ZAB (Figure S30). Galvanostatic discharge testing at 100 mA cm^−2^ revealed a specific capacity of 807 mAh g_Zn_^−1^ for Fe‐N_3_O@HPC, compared to 730 mAh g_Zn_^−1^ for Pt/C (Figure 5d). The simultaneous maintenance of higher discharge voltages in Fe‐N_3_O@HPC further highlights its dual advantage in energy density and power output per unit zinc consumption. Long‐term cycling stability was assessed through repeated charge‐discharge cycles at 10 mA cm^−2^ (Figure 5e). The Fe‐N_3_O@HPC system maintained a stable voltage gap of ∼1.4 V over 200 h, demonstrating exceptional durability. In contrast, the Pt/C‐IrO_2_ benchmark exhibited progressive voltage polarization and reduced cycle life, emphasizing the structural robustness of the single‐atom Fe catalyst design.

**FIGURE 5:**
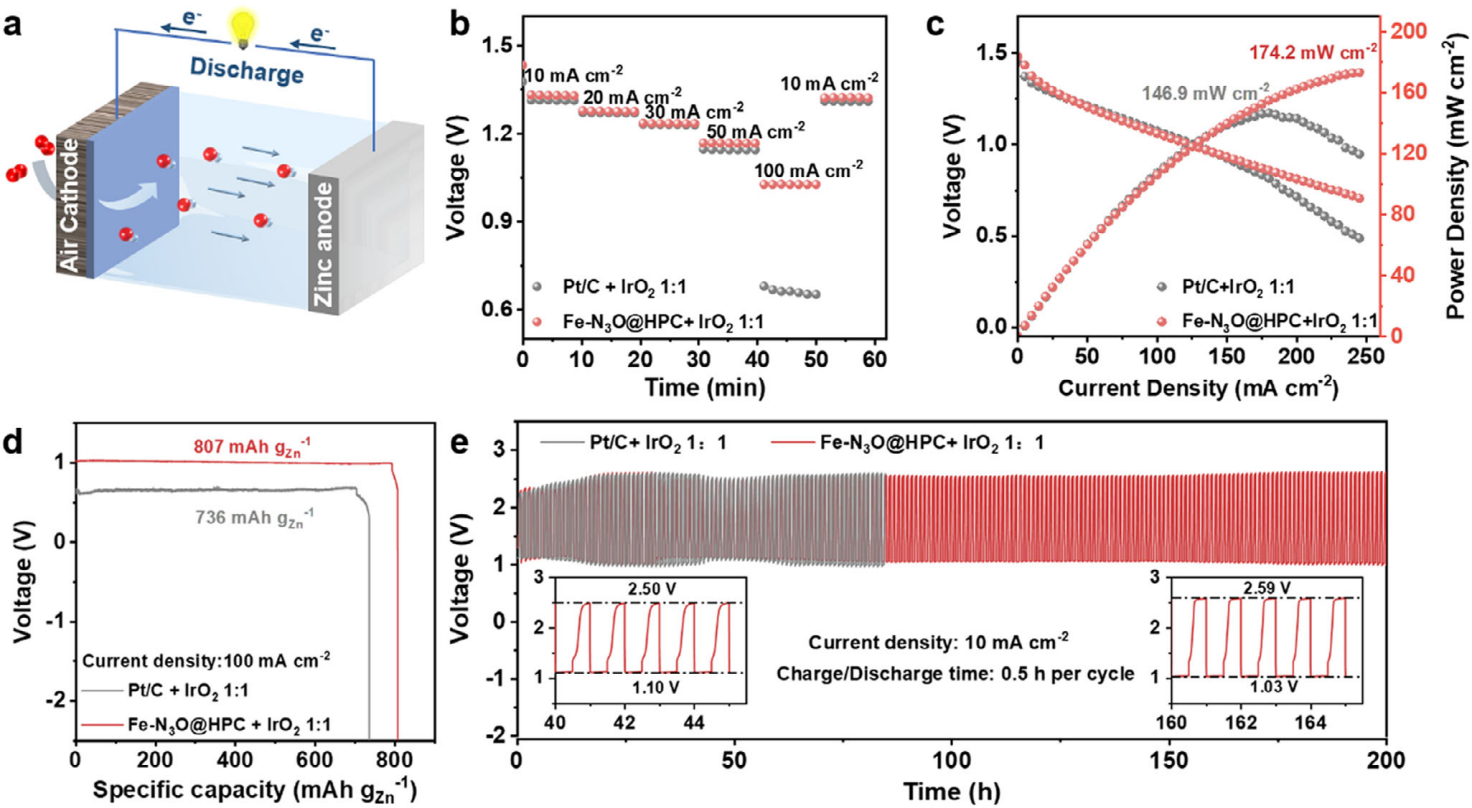
(a) Schematic illustration of ZAB device. (b) Galvanostatic discharge curves of the ZABs at various current densities. (c) Discharge and power density curves. (d) The specific capacities at 100 mA cm^−2^. (e) Long‐time galvanostatic discharge curves at 10 mA cm^−2^ for the ZABs.

## 3 Conclusion

In summary, we develop a facile Zn/MgO dual‐templating approach to fabricate high‐performance Fe SACs anchored on hierarchically porous lignin‐derived carbon. The oxygen‐rich lignin matrix serves a dual role as both carbon source and coordination modulator, enabling the formation of unique Fe‐N_3_O asymmetric active sites. The optimized catalyst achieves exceptional alkaline ORR performance, demonstrating a remarkable half‐wave potential (0.87 V vs. RHE) and stability (87% current retention after 30 h). When implemented in Zinc‐Air batteries, the catalyst delivers superior practical performance (174.2 mW cm^−2^ peak power density, 807 mAh g_Zn_^−1^ specific capacity), outperforming both leading non‐precious metal catalysts and commercial Pt/C benchmarks. Combination of operando spectroscopic studies and theoretical calculations indicates that the micropore‐dominated hierarchical architecture significantly enhances mass transport and oxygen adsorption kinetics. Meanwhile, compared to typical Fe‐N_4_ site, Fe‐N_3_O asymmetric geometry more pronounced electron depletion around Fe center and downshift the d‐band center, providing better ORR catalytic activity. This work establishes a novel paradigm for constructing asymmetric coordination SACs through biomass valorization, while advancing pore engineering strategies for electrocatalyst design.

## Author Contributions

**Gaixiu Yang** conceived and supervised the project. **Xuhong Zheng** performed the experiments and analyzed the data. **Yukun Luo** and **Mengxuan Zhang** assisted with materials synthesis, structural characterization, and electrochemical measurements. **Kang Xiao** and **Zhaoqing Liu** contributed to the theoretical calculations. **Xuebin Lv**, **Tianguang Wang**, **Jing Gu**, and **Haoran Yuan** helped analyze the experimental results. Gaixiu Yang and Xuhong Zheng linked the experiments and analysis, and all the authors discussed and wrote the manuscript.

## Conflicts of Interest

The authors declare no conflicts of interest.

## Supporting information




**Supporting File**: advs78503‐sup‐0001‐SuppMat.docx.

## Data Availability

The data that supports the findings of this study are available in the supplementary material of this article.
