# Supplementary material for: Oxygen‐Mediated Fe‐N3O Single‐Atom Sites Enabled by Lignin‐Derived Hierarchical Carbon for Efficient Oxygen Reduction
Source: Adv Sci (Weinh). 2026 Nov 9:e78503. Online ahead of print. doi: 10.1002/advs.78503 (PMC13748891; doi:10.1002/advs.78503)
Supplement: Supplementary file 1 [file ADVS-9999-e78503-s001.docx]

Supporting Information

**Oxygen-Mediated Fe-N_3_O Single-Atom Sites Enabled by Lignin-Derived Hierarchical Carbon for Efficient Oxygen Reduction**

*Xuhong Zheng ^b, c^, Yukun Luo ^b, c^, Mengxuan Zhang ^a, b,^ ^c^, Xuebin Lv ^a^, Tianguang Wang ^b, c^, Jing Gu ^b, c^, Kang Xiao ^d^, Zhaoqing Liu ^d^, Haoran Yuan ^b,^* *^c^ and Gaixiu Yang* *^a, b,^* *^c,^ **

^a^ School of Ecology and Environment, Xizang University, Lhasa 850000，PR China;

^b^ Guangzhou Institute of Energy Conversion, Chinese Academy of Sciences (CAS), Guangzhou 510640, PR China;

^c^ Guangdong Provincial Key Laboratory of High-Quality Recycling of End-of-Life New Energy Devices, Guangzhou 510640, PR China;

^d^ School of Chemistry and Chemical Engineering, Guangzhou University, Guangzhou 510006, China;

Corresponding Author: Prof. G.X. Yang

School of Ecology and Environment, Xizang University, Lhasa 850000, China
Guangzhou Institute of Energy Conversion, Chinese Academy of Sciences (CAS), Guangzhou 510640, PR China

Guangdong Provincial Key Laboratory of High-Quality Recycling of End-of-Life New Energy Devices, Guangzhou 510640, PR China

Email: yanggx@ms.giec.ac.cn

**1.Experimental Section**

**Materials**

All chemicals were used as received without further purification. Dealkaline lignin, zinc acetate dihydrate (Zn(CH_3_COO)_2_·2H_2_O, 99.5%), potassium hydroxide (KOH, AR), melamine (C_3_H_6_N_6_, 99%), nano magnesium oxide (nano MgO, 99.9%) and commercial 20% Pt/C were obtained from Shanghai Macklin Biochemical Co. Ltd (China). Iridium oxide (IrO_2_, 99.99%) was purchased from Shanghai Aladdin Co. Ltd. (China). Carbon cloth for Zn-air battery was provided by Suzhou Sinero Technology Co. Ltd. (China). Ferric nitrate hexahydrate (Fe (NO_3_)_3_⋅9H_2_O, AR) and ethanol absolute (AR) was supplied from Tianjin Damao Chemical Preparations Factory. Deionized water was used throughout all experiments.

**Preparation of Fe-N_3_O@HPC**

Fe-N_3_O@HPC was prepared through a pyrolysis process. In a typical synthesis, Zn (CH_3_COO)_2_ (0.438 g) and Fe (NO_3_)_3_ solution (200 μL, 0.5 M) were dispersed into deionized water (60 mL) to form solution. Subsequently, lignin (0.4 g), MgO template (0.3 g) and melamine (0.4 g) were added to the aforementioned solution and thoroughly dispersed. Thereafter, the resulting mixture was transferred to a thermostat water bath and stirred at 75 ℃ until complete solvent evaporation. The obtained solid (Fe/Zn-Lignin-MgO complex) was further dried overnight in a vacuum oven at 60 ℃. After being dried, the solid was collected as precursor and heated to 910 ℃ for 1 h (5 °C min^-1^) in a N_2_-filled tube furnace. The as-obtained powder was then etched in 1 M HCl solution at room temperature for 8 h to remove unwanted Fe nanoparticles and MgO templates. The final product was denoted as Fe-N_3_O@HPC.

**Preparation of Fe-N/O@Meso/Macro-PC**

Similar to the synthesis procedure of Fe-N_3_O@HPC, MgO was selected as the only template. lignin (0.4 g), melamine (0.4 g), MgO (0.3 g) and Fe (NO_3_)_3_ solution (200 μL, 0.5 M) were added into deionized water (60 mL) without Zn (CH_3_COO)_2_. The obtained precursors were then heated at a temperature of 910 °C for 1 h with a heating rate of 5 °C min^-1^ under N_2_ atmosphere. The as-prepared catalysts were labeled as Fe-N/O@Meso/Macro-PC.

**Preparation of Fe-N/O@Micro-PC**

The preparation process of Fe-N/O@Micro-PC was similar to Fe-N_3_O@HPC, except that Zn (CH_3_COO)_2_ was selected as the only template. Lignin (0.4 g), melamine (0.4 g), Zn (CH_3_COO)_2_ (0.438 g) and Fe (NO_3_)_3_ solution (200 μL, 0.5 M) were added into deionized water (60 mL) without MgO. The obtained precursors were then heated at a temperature of 910 °C for 1 h with a heating rate of 5 °C min^-1^ under N_2_ atmosphere. The as-prepared catalysts were labeled as Fe-N/O@Micro-PC.

**Preparation of Fe-N/O@PC**

The preparation process of Fe-N/O@PC was similar to Fe-N_3_O@HPC, except that 0.4 g Lignin, 0.4 g melamine and Fe (NO_3_)_3_ solution (200 μL, 0.5 M) were added into deionized water (60 mL) without MgO and Zn (CH_3_COO)_2_. The obtained precursors were then heated at a temperature of 910 °C for 1 h with a heating rate of 5 °C min^-1^ under N_2_ atmosphere. The as-prepared catalysts were labeled as Fe-N/O@PC.

**Characterizations**

Powder X-Ray diffraction (PXRD) analysis was conducted on a diffractometer (PANalytical, Holland) using Cu Kα radiation (40 kV, 40 mA, *λ*= 1.54184 Å) at room temperature. The morphology and structure of the catalysts were characterized using analytical field emission scanning electron microscopy (SEM, Hitachi SU-70, Japan), transmission electron microscopy (TEM, JEOL JEM-2100F, Japan), aberration-corrected high-angle annular dark-field scanning transmission electron microscope (AC-HAADF-STEM, JEOL JEM-ARM200F, Japan) and atomic force microscope (AFM, Bruker Multimode 8, Germany). Elemental distribution was assessed via energy-dispersive X-ray spectroscopy (EDS, JEOL, Japan). Elemental species were analyzed by X-ray photoelectron spectroscopy (XPS, Thermo Fisher Scientific Inc ESCALAB 250Xi, America). Raman spectroscopy was performed on LabRAM HR800 spectrometer (Horiba, France) and Fourier transform infrared (FT-IR) spectroscopy was carried out on a Nicolet iS50 spectrometer (Thermo Fisher Scientific Inc, America). Nitrogen adsorption/desorption isotherms were measured at 77 K using an ASAP 2460 (Micromeritics, America). The surface area was evaluated by the Brunauer-Emmett-Teller (BET) method. Pore size distribution was analyzed from the adsorption branch by DFT method over a relative pressure (P/P_0_) range from 1×10^-7^ to 1. Inductively coupled plasma-Mass Spectrometry (ICP-MS, Agilent 7900, Agilent Technologies Inc., America) was used to quantify the amount of Fe in the samples. The X-ray absorption fine structure (XAFS) analysis was carried on beamline 1W2A at the Beijing Synchrotron Radiation Facility (BSRF, Beijing, China). The acquired XAFS data were processed using standard procedures within the Athena module of the IFEFFIT software package and the fitting of EXAFS data were performed using the Artemis module^[1]^. The wavelet transforms of EXAFS data were performed by the Morlet wavelet using HAMA analysis software^[2]^.

**Electrochemical measurements**

Electrochemical measurements were performed at room temperature on a CHI760e electrochemical workstation employing a standard three-electrode system. A rotating disk electrode (RDE) or a rotating ring-disk electrode (RRDE) was used as working electrode (5 mm in diameter). A graphite rod and a Hg/HgO electrode were employed as the counter electrode and reference electrode, respectively. The electrolyte was N_2_ -/O_2_-saturated 0.1 M KOH aqueous solution. To prepare the catalyst ink, catalyst powder (5 mg) was dispersed in 1.0 mL mixture solvent containing ethanol (490 μL), deionized water (490 μL) and 5 wt% Nafion solution (20 μL). Subsequently, the catalyst ink (10 µL) was uniformly deposited onto the working electrode. Before the ORR tests, cyclic voltammetry (CV) was performed in N_2_-saturated electrolyte solution at a scan rate of 50 mV s^−1^ for at least 5 cycles to clean and stabilize the catalyst surface. Then the Linear sweep voltammetry (LSV) measurements were carried out from -0.8 to 0.3 V vs. Hg/HgO reference electrode at a scan rate of 10 mV s^−1^ and a rotation rate of 1600 rpm without IRs compensations. The durability of the catalyst was evaluated by the Chronoamperometric (CA) method in O_2_-saturated 0.1 M KOH at a constant potential of -0.465 V vs. Hg/HgO. RRDE measurements were conducted under identical conditions to the LSV measurements while the ring electrode was held at 0.5 V vs. Hg/HgO reference electrode. The H_2_O_2_ yield was calculated from the ring current (I_r_) and the disk current (I_d_) using the following equations:

$$\text{H}\text{2}\text{O}\text{2 }\text{\%} =\text{200}\times\frac{\text{I}\text{r}\text{ }\text{/ N}}{\text{I}\text{d}+\text{I}\text{r }\text{/ N}}$$

$$\text{n} =\text{4}\times\frac{\text{I}\text{d }}{\text{I}\text{d}+\text{I}\text{r }\text{/ N}}$$

where *I_d_* is the Faradaic current at the disk, *I_r_* is the Faradaic current at the ring, *n* is the transferred electron number and *N* is the H_2_O_2_ collection coefficient at the ring, which was 0.37 after calibration by [Fe(CN)_6_] ^3-/4-^ redox system^[3]^.

The number of electrons transferred during ORR was also calculated at different rotating rates (625-2500 rpm) according to Koutecky–Levich (K-L) equation:

$$\frac{\text{1}}{\text{j}}\text{=}\frac{\text{1}}{\text{j}_{\text{L}}}\text{+}\frac{\text{1}}{\text{j}_{\text{k}}}\text{=}\frac{\text{1}}{\text{B}\text{ω}^{\frac{\text{1}}{\text{2}}}}\text{+}\frac{\text{1}}{\text{j}_{\text{k}}}$$

$$\text{B=}\text{0.62}\text{nF}\text{C}_{\text{0}}\text{D}_{\text{0}}^{\text{2/3}}\text{v}^{\text{-1/6}}$$

where *j*, *j_L_*, and *j_k_* are the measured current density, limiting current density and kinetic current density, respectively. *ω* is the angular velocity of rotating disk electrode, *F* is Faraday constant (96485 C mol^−1^), *C_0_* is the bulk concentration of O_2_ (1.2 × 10^−6^ mol cm^−3^), *D_0_* is the diffusion coefficient (1.9 × 10^−5^ cm^2^ s^−1^) of O_2_ in 0.1 M KOH solution.

The Fe mass activity (*MA*) was calculated by normalizing the kinetic current density based on the following equations:

$$\text{MA=}\frac{\text{j}_{\text{k}}}{\text{C}_{\text{cat}}\text{W}_{\text{Fe}}}$$

where *C_cat_* is the catalyst loading (0.25 mg cm^-2^) and *W_Fe_* represents the weight percentage of Fe measured by ICP-MS.

All potentials obtained using Hg/HgO as the reference electrode were converted to RHE according the equation:

*E*_𝑅𝐻𝐸_ = 𝐸*_Hg/HgO_* + 0.059*pH* + 𝐸^θ^*_Hg/HgO_*

where 𝐸*_Hg/HgO_* is the measured potential and 𝐸^θ^*_Hg/HgO_=*0.098 V vs. RHE.

**Construction of Zn-Air Battery**

The aqueous Zn-air batteries were assembled with a carbon cloth coated with the catalyst ink as an air cathode. ORR catalyst (3 mg) and IrO_2_ (3 mg) powder was mixed and dispersed in 320 μL solvent containing ethanol (150 μL), deionized water (150 μL) and 5 wt% Nafion solution (20 μL) to form catalyst ink. After sonication for 1 h, 320 μL catalyst ink was uniformly coated onto a commercial carbon cloth substrate (6 cm×2.5 cm). The coated substrate was then dried in an oven at 60 ℃ to obtain a gas diffusion electrode (GDE) with a geometric area of 1 cm^2^. The gas diffusion electrode (GDE) served as the air cathode of the Zn-air battery, while a zinc sheet was used as the anode, and 6 M KOH was employed as the electrolyte. Battery performances were evaluated using a CHI760e electrochemical workstation and a Neware battery test system. The charge-discharge cycle stability was carried out at room temperature, employing a cycling protocol of 30 minutes charging followed by 30 minutes discharging per cycle (total 1 hour per cycle).

**Operando electrochemical impedance spectroscopy (EIS)** **measurements**

The operando electrochemical impedance spectroscopy (EIS) tests were performed with catalyst-coated carbon cloth in 0.1 M O_2_-saturated KOH solution by an electrochemical workstation CHI760E with a standard three-electrode electrochemical cell. The measured potential ranges of the EIS were 0.6-1.0 V vs. RHE in the frequency range of 0.01-1000000 Hz. Before the EIS spectrum was collected at each test voltage, the same voltage was applied to the catalyst electrode for 10 minutes through the i-t test. To ensure full exposure of the active sites, the catalyst ink was prepared by dispersing catalyst (5 mg) into 1.0 mL of the mixed solvent containing deionized water (490 μL), ethanol (490 μL) and 5 wt% Nafion solution (20 μL) by continuous ultrasonication for 30 min. 20 μL catalyst ink was dropped onto carbon cloth as the working electrode. And a graphite rod and a Hg/HgO electrode were employed as the counter electrode and reference electrode, respectively.

The EIS data was fitted using ZView2, which is a series circuit composed of the solution resistance (R_s_), charge transfer resistance (R_ct_), mass transfer resistance (R_mt_) and the constant phase element (CPE). And the distribution of relaxation times (DRT) analysis was also performed, which is a model-free analysis designed for complex electrochemical systems:

$$z\left( f \right)=R_{\infty}+R_{pol}\int_{0}^{\infty} \frac{g(\tau)}{1+2i\pi f\tau}d\tau$$

where $R_{\infty}$ represents the ohmic resistance, $R_{pol}$ corresponds to the polarization resistance, while *τ* and $f$ denote the relaxation time and frequency, respectively.

DRT analysis from EIS data were calculated by MATLAB with a toolbox of DRT-TOOLS developed by the research group of Professor Francesco Ciucci. The regularization parameter λ is set to 10^-3^ for the Tikhonov regularization approach^[4]^.

**In-situ Raman Measurements**

In-situ Raman measurements were performed in a three-electrode in-situ Raman epoxy pool cell using a LabRAM HR800 spectrometer system (Horiba, France). Catalyst ink (10 µL) was sprayed on the glassy carbon electrode (5 mm in diameter) to form active area. Glassy carbon electrode loaded with catalyst was used as the working electrode. A Pt wire was used as the counter electrode and an Ag/AgCl electrode was used as the reference electrode. The electrolyte was O_2_-saturated 0.1 M KOH. 532 nm laser (25%) was focused on the sample surface without contacting the electrolyte.

The potentials obtained using Ag/AgCl electrode as the reference electrode were converted to RHE according the Nernst equation:

$$E_{RHE}=E_{Ag/AgCl}+0.059pH+E_{Ag/AgCl}^{\theta}$$

where $E_{Ag/AgCl}$ is the measured potential and $E_{Ag/AgCl}^{\theta}$=0.1976 V vs. RHE.

**Theoretical Calculations**

Density functional theory (DFT) calculations were conducted by the Vienna Ab initio Simulation Package (VASP)^[5]^. Perdew-Burke-Ernzerhof (PBE) generalized gradient approximation (GGA) was used to describe the exchange-correlation potential^[6]^. And the interactions between atomic cores and valence electrons were accounted by the projector augmented wave (PAW)^[7]^. All calculations were spin-polarized. The wave functions of valence electrons were expanded using a plane wave basis set with a cut-off energy of 400 eV, which is large enough to avoid the problems of Pulay stress and changes. The van der Waals (Vdw) interactions between atoms were described by DFT-D3 method^[8]^. The energy and force convergence criteria of the self-consistent iteration were set to 10^-4^ eV and 0.05 eV Å^-1^, respectively, for geometrically optimizing the calculation models. Brillouin zone sampling was conducted using a 2×2×1 Gamma centered Monkhorst-Pack grids k-points.

The alkaline ORR reaction process consists of four steps as follows:

*+O_2_ + H_2_O + e^-^ → *OOH + OH^-^

*OOH + e^-^ → *O + OH^-^

*O + H_2_O + e^-^ → *OH + OH^-^

OH* + e^-^ → OH^-^+ *

In our study, the Gibbs free energy change (Δ*G*) for the proton-coupled electron transfer (PCET) step was calculated using the computational hydrogen electrode (CHE) model. This model equates one-half of the chemical potential of the hydrogen as the chemical potential of the proton-electron pair. Based on this principle, Δ*G* was determined using the following equation:

Δ*G*= Δ*E* + Δ*ZPE* – *T*Δ*S*

where Δ*E* represents the electronic energy difference between the initial and final states of the PCET step. Δ*ZPE* is the zero-point energy difference between the initial and final states. *T*Δ*S* represents the entropy difference between the initial and final states, where *T* is the absolute temperature.

**2.Supplementary Figures and Tables**

**Figure S1.** SEM images of (a, b) lignin, (c, d) MgO template, (e, f) complex precursor before carbonization, (g, h) Fe-N/O@PC.

**Figure S2.** (a) Nitrogen adsorption and desorption isotherms measured at 77 K with (b) corresponding pore size distribution, (c) BET surface area and (d) pore volume.

**Figure S3.** XRD patterns of Fe-N_3_O@HPC, Fe-N/O@Meso/Macro-PC, Fe-N/O@Micro-PC and Fe-N/O@PC.

**Figure S4.** Raman spectra of Fe-N_3_O@HPC, Fe-N/O@Meso/Macro-PC, Fe-N/O@Micro-PC and Fe-N/O@PC.

**Figure S5.** HR-TEM image of the Fe-N_3_O@HPC.

**Figure S6.** (a, b) TEM images, (c) HR-TEM image, and (d) EDS elemental mapping of the Fe-N/O@Meso/Macro-PC.

**Figure S7.** (a, b) TEM images, (c) HR-TEM image, (d) EDS elemental mapping of the Fe-N/O@Micro-PC.

**Figure S8.** (a, b) TEM images, (c) HR-TEM image and (d) EDS elemental mapping of the Fe-N/O@PC.

**Figure S9.** The XPS survey spectra of (a) Fe-N_3_O@HPC, (b) Fe-N/O@Meso/Macro-PC, (c) Fe-N/O@Micro-PC and (d) Fe-N/O@PC.

**Figure S10.** High-resolution Fe 2*p* XPS spectra of the Fe-N_3_O@HPC, Fe-N/O@Meso/Macro-PC, Fe-N/O@Micro-PC, Fe-N/O@PC.

**Figure S11.** High-resolution N 1*s* XPS spectra of (a) Fe-N/O@Meso/Macro-PC, (b) Fe-N/O@Micro-PC and (c) Fe-N/O@PC with (d) the corresponding proportions of N species.

**Figure S12.** High-resolution O 1*s* XPS spectra of (a) Fe-N_3_O@HPC, (b) Fe-N/O@Meso/Macro-PC, (c) Fe-N/O@Micro-PC and (d) Fe-N/O@PC.

**Figure S13.** FT-IR spectra of the Fe-N_3_O@HPC, Fe-N/O@Meso/Macro-PC, Fe-N/O@Micro-PC, Fe-N/O@PC.

**Figure S14.** Fe oxidation state determined from Fe K-edge XANES derivative spectra.

**Figure S15.** ORR CV curves in O_2_- and N_2_-saturated 0.1 M KOH.

**Figure S16.** Fe mass-normalized kinetic current density for the as-prepared catalysts at 0.8 V vs RHE

**Figure S17.** CV curves at different scan rates for (a) Fe-N_3_O@HPC, (b) Fe-N/O@Meso/Macro-PC, (c) Fe-N/O@Micro-PC, (d) Fe-N/O@PC.

**Figure S18**. (a) Polarization curve of RRDE and (b) the correction of collection efficiency in the solution of 1 mM K_3_Fe(CN)_6_ and 0.1 M KOH.

**Figure S19.** ORR polarization curves (a, c, e, g, i) at different rotating speeds and the corresponding K-L plots (b, d, f, h, j) for Fe-N_3_O@HPC, Fe-N/O@Meso/Macro-PC, Fe-N/O@Micro-PC, Fe-N/O@PC, Pt/C.

**Figure S20.** ORR polarization plots of Fe-N_3_O@HPC with and without 10 mM KSCN in O_2_-saturated 0.1 m KOH.

**Figure S21.** (a) XPS spectra (b)N 1*s* (c) Fe 2*p* and (d) O 1*s* spectra of Fe-N_3_O@HPC before and after reaction.

**Figure S22.** LSV curves of (a) Pt/C and (b) Fe-N_3_O@HPC before and after 8000 cycles at the range of 0.6–1.0 V.

**Figure S23.** (a-d) the fitting equivalent circuit model and (e-h) the fitting results of Nyquist plots for Fe-N_3_O@HPC, Fe-N/O@Meso/Macro-PC, Fe-N/O@Micro-PC and Fe-N/O@PC.

**Figure S24.** The (a) R_mt_ value curves and (b) R_ct_ value curves at 1.0−0.6 V vs RHE of Fe-N_3_O@HPC, Fe-N/O@Meso/Macro-PC and Fe-N/O@Micro-PC.

**Figure S25.** DRT plots for (a) Fe-N_3_O@HPC, (b) Fe-N/O@Meso/Macro-PC, (c) Fe-N/O@Micro-PC and (d) Fe-N/O@ PC.

**Figure S26.** Optimized configuration for (a) Fe-N_4_ and (b) Fe-N_3_O. Gray, red, blue, and yellow balls represent C, O, N and Fe atoms, respectively.

**Figure S27.** ORR mechanism in alkaline solution. Gray, red, white, and yellow balls represent C, O, H and Fe atoms, respectively.

**Figure S28.** ORR free energy diagrams under U=0 V.

**Figure S29.** The open circuit potential (OCP) of (a) Pt/C and (b) Fe-N_3_O@HPC in primary Zn-air batteries.

**Figure S30.** Photos of ZAB of (a) before and (b) after powering.

**Table S1**. Fe contents of synthesized catalysts determined by ICP-MS (weight ratio, wt%)

| **Sample** | **Fe content (wt%)** |
| --- | --- |
| Fe-N_3_O@HPC | 0.5841 |
| Fe-N/O@Meso/Macro-PC | 0. 5989 |
| Fe-N/O@Micro-PC | 0.7768 |
| Fe-N/O@PC | 1.4818 |

**Table S2**. EXAFS data fitting parameters at the Fe K-edge for samples

| **Sample** | **Path** | **CN** | **R (Å)** | **σ^2^ (Å)** | **ΔE_0_ (eV)** | **R factor** |
| --- | --- | --- | --- | --- | --- | --- |
| Fe foil | Fe-Fe1  Fe-Fe2 | 8  6 | 2.48±0.02  2.85±0.02 | 0.0058  0.0059 | 5.34±2.63 | 0.0055 |
| Fe-N_3_O@HPC | Fe-N  Fe-O | 3.32  1.25 | 2.08±0.01  1.87±0.05 | 0.0012  0.0011 | 6.41±1.45 | 0.0181 |

k range: 3-13 (Å^-1^); R range: 1-2.4 Å; S_0_^2^= 0.868 was determined from the Fe foil and used for Fe K-edge fitting. A reasonable range of EXAFS fitting parameters: CN > 0; σ^2^ > 0 Å^2^; R factor ≤ 0.02

**Table S3.** Fe contents of electrolyte after reaction and catalysts before reaction

| **electrolyte** | **Fe-N_3_O@HPC** | **Fe loss percentage** |
| --- | --- | --- |
| 1.097 μg_Fe_ L^-1^ | 0.5841 mg_Fe_ g_cat_^-1^ | 2.5 % |

**Table S4.** Performance comparison of some recently reported PGM-free catalysts for ORR in 0.1 M KOH solution.

| **Sample** | **Onset potential**  **(V vs. RHE)** | **Half-wave**  **potential**  **(V vs. RHE)** | **References** |
| --- | --- | --- | --- |
| Fe-N_3_O@HPC | 0.98 | 0.87 | This work |
| Pt/C | 0.95 | 0.83 | This work |
| Zn-CoFe@NLC | 0.99 | 0.84 | [9] |
| NPS-HPCNF | N.A. | 0.86 | [10] |
| MF_0.04_/CN | 0.95 | 0.79 | [11] |
| Mn–S_1_O_4_G-600 | 0.98 | 0.86 | [12] |
| NiCo_1.8_Fe_0.2_O_4_@NCF | 0.99 | 0.86 | [13] |
| SA-Fe-SNC@900 | 1.01 | 0.876 | [14] |
| VP/CNs | 1.08 | 0.86 | [15] |
| Fe SA/NCZ | 1.06 | 0.87 | [16] |
| FeSA/N-PSCS | 0.976 | 0.87 | [17] |
| meso-Fe-N-C | N.A. | 0.846 | [18] |
| Fe SA-NSC-900 | 0.94 | 0.86 | [19] |
| MoS_2_@Fe-N-C NSs | N.A. | 0.84 | [20] |
| Co-pyridinic N-C | 0.99 | 0.87 | [21] |
| Fe/OES | 1 | 0.85 | [22] |

**Table S5.** Performance comparison of various PGM-free catalysts in aqueous Zn-air batteries.

| **Sample** | **Peak power**  **density**  **(mW cm^-2^)** | **Specific**  **capacity**  **(mAh g_Zn_^-1^)** | **References** |
| --- | --- | --- | --- |
| Fe-N_3_O@HPC | 174.2 | 807@100 mA cm^-2^ | This work |
| Zn-CoFe@NLC | 164 | 770@10 mA cm^-2^ | [9] |
| Ce_0.05_-N-C/Cu_clu_ | 102 | 729.9@20 mA cm^-2^ | [23] |
| MF_0.04_/CN | 138 | 742.8@100 mA cm^-2^ | [11] |
| Fe/Se-NC | 137.1 | 818.9@10 mA cm^-2^ | [24] |
| Fe_SA/AC_@HNC | 171.5 | 811.8@20 mA cm^-2^ | [25] |
| CoP_3_/CeO_2_/C-2 | 150 | 768@5 mA cm^-2^ | [26] |
| Fe-SA@PNC | 149 | 749@10 mA cm^-2^ | [27] |
| NP-Co_SA_NC | 158.1 | 768.4@20 mA cm^-2^ | [28] |
| Eu_2_O_3_-Co/NC | 123.3 | 760.7 @5 mA cm^-2^ | [29] |
| FePC & rGO | 103 | 739.7@10 mA cm^-2^ | [30] |
| Co-CoN_4_@NCNs | 118.8 | 776.7@10 mA cm^-2^ | [31] |
| Fe_1_/*d*-CN | 144 | 770@10 mA cm^-2^ | [32] |
| Fe/N-CNRs | 181.8 | 771.7@10 mA cm^-2^ | [33] |
| Fe-N/P-C-700 | 133.2 | 723.6@100 mA cm^-2^ | [34] |
| Fe/Ni-Nx/OC | 148 | 712@50 mA cm^-2^ | [35] |

**3.References in Supporting Information**

[1]	B. Ravel, and M. Newville, "ATHENA, ARTEMIS, HEPHAESTUS: Data Analysis for X-ray Absorption Spectroscopy Using IFEFFIT," *Journal of Synchrotron Radiation* 12 (2005): 537-541, <https://doi.org/10.1107/s0909049505012719>

[2]	H. Funke, M. Chukalina, and A. Rossberg, "Wavelet Analysis of Extended X-ray Absorption Fine Structure Data," *Physica Scripta* T115 (2005): 232-234

[3]	Z. T. Zhang, W. B. Chen, H. K. Chu, et al., "Fe-O_4_ Motif Activated Graphitic Carbon via Oxo-Bridge for Highly Selective H_2_O_2_ Electrosynthesis," *Angewandte Chemie International Edition* 63 (2024): e202410123, <https://doi.org/10.1002/anie.202410123>

[4]	X. Q. Wei, M. An, X. N. Tang, et al., "Near-100% Site Utilization of Single Atoms for Efficient Electrocatalysis," *Nature Communications* 17 (2026): 1013 <https://doi.org/10.1038/s41467-025-67756-8>

[5]	G. Kresse, and J. Hafner, "Ab Initio Molecular-Dynamics for Liquid-Metals," *Physical Review B* 47 (1993): 558-561, <https://doi.org/10.1103/PhysRevB.47.558>

[6]	J. P. Perdew, K. Burke, and M. Ernzerhof, "Generalized Gradient Approximation Made Simple," *Physical Review Letters* 77 (1996): 3865-3868, <https://doi.org/10.1103/PhysRevLett.77.3865>

[7]	G. Kresse, and D. Joubert, "From Ultrasoft Pseudopotentials to the Projector Augmented-Wave Method," *Physical Review B* 59 (1999): 1758-1775, <https://doi.org/10.1103/PhysRevB.59.1758>

[8]	S. Grimme, J. Antony, S. Ehrlich, and H. Krieg, "A Consistent and Accurate Ab Initio Parametrization of Density Functional Dispersion Correction (DFT-D) for the 94 Elements H-Pu," *Journal of Chemical Physics* 132 (2010): 154104, <https://doi.org/10.1063/1.3382344>

[9]	J. H. Zhang, J. L. Liu, D. L. Chen, et al., "Zn Volatilization-Induced Structural Evolution in Lignin-Derived Co/Fe Heterojunction Catalysts Enables Long-Life Zinc-Air Batteries," *Applied Catalysis B-Environment and Energy* 386 (2026): 126386, <https://doi.org/10.1016/j.apcatb.2026.126386>

[10]	X. Xu, H. L. Wu, Y. Yan, et al., "Electronic Structure Modulation in N, P, S Tri-Doped Nanofibers with Interpenetrated Pores for Enhanced Oxygen Reduction Reaction," *Advanced Energy Materials* 15 (2025): 2405236, <https://doi.org/10.1002/aenm.202405236>

[11]	M. Dan, X. T. Zhang, C. Y. Du, et al., "Atomically Dispersed Fe Confined into MnO Nanoclusters Enhances Alkaline Oxygen Reduction Activity and Stability," *Angewandte Chemie International Edition* 64 (2025): e202501531, <https://doi.org/10.1002/anie.202501531>

[12]	X. Bai, Y. Wang, J. Y. Han, et al., "Double Coordination Shell Modulation of Nitrogen-Free Atomic Manganese Sites for Enhancing Oxygen Reduction Performance," *Chemical Science* 15 (2024): 19466-19472, <https://doi.org/10.1039/d4sc05998k>

[13]	Y. Y. Liu, L. M. Zhou, S. L. Liu, et al., "Fe, N-Inducing Interfacial Electron Redistribution in NiCo Spinel on Biomass-Derived Carbon for Bi-functional Oxygen Conversion," *Angewandte Chemie International Edition* 63 (2024): e202319983, <https://doi.org/10.1002/anie.202319983>

[14]	Z. H. Chen, X. W. Peng, Z. X. Chen, et al., "Mass Production of Sulfur-Tuned Single-Atom Catalysts for Zn-Air Batteries," *Advanced Materials* 35 (2023): 202209948, <https://doi.org/10.1002/adma.202209948>

[15]	H. Y. Xia, R. Y. Pang, X. Y. M. Dong, et al., "Boosting Oxygen Reduction Reaction Kinetics by Designing Rich Vacancy Coupling Pentagons in the Defective Carbon," *Journal of the American Chemical Society* 145 (2023): 25695-25704, <https://doi.org/10.1021/jacs.3c08556>

[16]	C. L. Jiao, Z. Xu, J. Z. Shao, et al., "High-Density Atomic Fe-N_4_/C in Tubular, Biomass-Derived, Nitrogen-Rich Porous Carbon as Air-Electrodes for Flexible Zn-Air Batteries," *Advanced Functional Materials* 33 (2023): 2213897, <https://doi.org/10.1002/adfm.202213897>

[17]	M. X. Shen, J. Liu, J. Li, et al., "Breaking the N-limitation with N-Enriched Porous Submicron Carbon Spheres Anchored Fe Single-Atom Catalyst For Superior Oxygen Reduction Reaction And Zn-Air Batteries," *Energy Storage Materials* 59 (2023): 102790, <https://doi.org/10.1016/j.ensm.2023.102790>

[18]	Y. Zhou, Y. N. Yu, D. S. Ma, et al., "Atomic Fe Dispersed Hierarchical Mesoporous Fe-N-C Nanostructures for an Efficient Oxygen Reduction Reaction," *ACS Catalysis* 11 (2021): 74-81, <https://doi.org/10.1021/acscatal.0c03496>

[19]	M. R. Wang, W. J. Yang, X. Z. Li, et al., "Atomically Dispersed Fe-Heteroatom (N, S) Bridge Sites Anchored on Carbon Nanosheets for Promoting Oxygen Reduction Reaction," *ACS Energy Letters* 6 (2021): 379-386, <https://doi.org/10.1021/acsenergylett.0c02484>

[20]	Y. Yan, S. Liang, X. Wang, et al., "Robust Wrinkled MoS_2_/N-C Bifunctional Electrocatalysts Interfaced with Single Fe Atoms for Wearable Zinc-Air Batteries," *Proceedings of the National Academy of Sciences of the United States of America* 118 (2021): e2110036118, <https://doi.org/10.1073/pnas.2110036118>

[21]	Y. Ha, B. Fei, X. X. Yan, et al., "Atomically Dispersed Co-Pyridinic N-C for Superior Oxygen Reduction Reaction," *Advanced Energy Materials* 10 (2020): 2002592, <https://doi.org/10.1002/aenm.202002592>

[22]	C. C. Hou, L. L. Zou, L. M. Sun, et al., "Single-Atom Iron Catalysts on Overhang-Eave Carbon Cages for High-Performance Oxygen Reduction Reaction," *Angewandte Chemie International Edition* 59 (2020): 7384-7389, <https://doi.org/10.1002/anie.202002665>

[23]	C. G. Zhang, Q. L. Zhao, J. F. Wu, et al., "Ce Single-Atoms Modulation of Cu Atomic Clusters for Enhanced Oxygen Reduction and Durable Zn-Air Batteries," *Advanced Functional Materials* 36 (2026): e15095, <https://doi.org/10.1002/adfm.202515095>

[24]	W. C. Zhang, X. Z. Li, S. H. Guo, et al., "Long-Range Electronic Modulation of Fe Atomic Sites via Third Coordination Layer Selenium Anchoring for Enhanced Oxygen Reduction Reaction in Zn-Air and Mg-Air Batteries," *Chemical Engineering Journal* 521 (2025): 167173, <https://doi.org/10.1016/j.cej.2025.167173>

[25]	H. W. Zhang, H. C. Chen, S. Feizpoor, et al., "Tailoring Oxygen Reduction Reaction Kinetics of Fe-N-C Catalyst via Spin Manipulation for Efficient Zinc-Air Batteries," *Advanced Materials* 36 (2024): 2400523, <https://doi.org/10.1002/adma.202400523>

[26]	J. M. Li, Y. M. Kang, Z. Q. Lei, and P. Liu, "Well-Controlled 3D Flower-Like CoP_3_/CeO_2_/C Heterostructures as Bifunctional Oxygen Electrocatalysts for Rechargeable Zn-Air Batteries," *Applied Catalysis B-Environment and Energy* 321 (2023): 122029, <https://doi.org/10.1016/j.apcatb.2022.122029>

[27]	M. X. Shen, J. L. Qi, K. Gao, et al., "Chemical Vapor Deposition Strategy for Inserting Atomic FeN_4_ Sites into 3D Porous Honeycomb Carbon Aerogels as Oxygen Reduction Reaction Catalysts in High-Performance Zn-Air Batteries," *Chemical Engineering Journal* 464 (2023): 142719, <https://doi.org/10.1016/j.cej.2023.142719>

[28]	J. Rong, E. R. Gao, N. C. Liu, et al., "Porphyrinic MOF-Derived Rich N-Doped Porous Carbon with Highly Active CoN_4_C Single-Atom Sites for Enhanced Oxygen Reduction Reaction and Zn-Air Batteries Performance," *Energy Storage Materials* 56 (2023): 165-173, <https://doi.org/10.1016/j.ensm.2023.01.022>

[29]	X. Wang, J. W. Wang, P. Wang, et al., "Engineering 3d-2p-4f Gradient Orbital Coupling to Enhance Electrocatalytic Oxygen Reduction," *Advanced Materials* 34 (2022): 2206540, <https://doi.org/10.1002/adma.202206540>

[30]	Z. Y. Mei, S. Cai, G. F. Zhao, et al., "Understanding Electronic Configurarions and Coordination Environment for Enhanced ORR Process and Improved Zn-Air Battery Performance," *Energy Storage Materials* 50 (2022): 12-20, <https://doi.org/10.1016/j.ensm.2022.05.006>

[31]	K. X. Ding, J. G. Hu, J. Luo, et al., "Robust Electronic Correlation of Co-CoN_4_ Hybrid Active Sites for Durable Rechargeable Zn-Air Batteries," *Advanced Functional Materials* 32 (2022): 2207331, <https://doi.org/10.1002/adfm.202207331>

[32]	M. Q. Zhao, H. R. Liu, H. W. Zhang, et al., "A pH-Universal ORR Catalyst with Single-Atom Iron Sites Derived from a Double-Layer MOF for Superior Flexible Quasi-Solid-State Rechargeable Zn-Air Batteries," *Energy & Environmental Science* 14 (2021): 6455-6463, <https://doi.org/10.1039/d1ee01602d>

[33]	X. F. Gong, J. B. Zhu, J. Z. Li, et al., "Self-Templated Hierarchically Porous Carbon Nanorods Embedded with Atomic Fe-N_4_ Active Sites as Efficient Oxygen Reduction Electrocatalysts in Zn-Air Batteries," *Advanced Functional Materials* 31 (2021): 2008085, <https://doi.org/10.1002/adfm.202008085>

[34]	K. Yuan, D. Lützenkirchen-Hecht, L. B. Li, et al., "Boosting Oxygen Reduction of Single Iron Active Sites via Geometric and Electronic Engineering: Nitrogen and Phosphorus Dual Coordination," *Journal of the American Chemical Society* 142 (2020): 2404-2412, <https://doi.org/10.1021/jacs.9b11852>

[35]	Z. J. Zhu, H. J. Yin, Y. Wang, et al., "Coexisting Single-Atomic Fe and Ni Sites on Hierarchically Ordered Porous Carbon as a Highly Efficient ORR Electrocatalyst," *Advanced Materials* 32 (2020): 2004670, <https://doi.org/10.1002/adma.202004670>
